# Exploring the structure of college students' adaptability by using cross‐lagged path analysis: The role of emotional adaptability

**DOI:** 10.1002/pchj.721

**Published:** 2023-12-27

**Authors:** Shixiu Ren, Shunxin Ji, Xinyang Liu, Tour Liu

**Affiliations:** ^1^ Collaborative Innovation Center of Assessment Toward Basic Education Quality Beijing Normal University Beijing China; ^2^ Faculty of Psychology Tianjin Normal University Tianjin China; ^3^ Key Research Base of Humanities and Social Sciences of the Ministry of Education, Academy of Psychology and Behavior Tianjin Normal University Tianjin China; ^4^ Center of Collaborative Innovation for Assessment and Promotion of Mental Health Tianjin Normal University Tianjin China

**Keywords:** adaptability, emotional adaptability, path analysis model, stressor, structural equation modeling

## Abstract

Adaptability is an important psychological trait for college students. However, the components of adaptability contained significant inconsistencies in previous studies. On the one hand, there were discrepancies among the adaptability dimensions. On the other hand, significant inconsistencies were found in the connections among different aspects of adaptability. Therefore, the current research aimed to investigate the latent relationship among various components of adaptability. To achieve this, 565 volunteers were recruited to complete a 5‐min cross‐sectional survey. Subsequently, 402 participants were recruited to complete an 8‐min longitudinal survey. The current study comprised two sub‐studies: Study 1 utilized a structural equation model to examine the relationship between various dimensions of adaptability in a cross‐sectional dataset, while Study 2 employed the cross‐lagged panel model to validate the latent relationship between emotional adaptability and other types of adaptability using a longitudinal dataset. Results from the cross‐sectional study indicated significant associations between emotional adaptability and other types of adaptability, with coefficients ranging from .231 to .588. The longitudinal study revealed that emotional adaptability at Time 2 and 3 could be predicted by learning adaptability, professional adaptability, and economic adaptability at Time 1 and 2. Consequently, the research concluded that individuals' emotional maladjustment could be predicted by maladaptive difficulties in learning, professional settings, homesickness, interpersonal relationships, and economics.

## INTRODUCTION

Adaptability, as described by the Council of Chief State School Officers (CCSSO), is a crucial psychological trait for individual performance in both educational and professional settings (College and Career Competency Framework, 2016; Holliman‐Andrew et al., 2021). It facilitates students' transition from being a school student to becoming a social citizen with high levels of competency (Elena et al., 2021; Sima et al., 2021). Thus, many researchers believed that adaptability is a soft skill that enables individuals to quickly learn new abilities and behaviors in response to uncertain, unfamiliar, or rapidly changing circumstances (Cao & Mao, 2008; Luo, 2020; Martin et al., 2013). As society rapidly evolves, students are increasingly required to exhibit higher levels of adaptability (Quintas‐Hijos et al., 2020). Consequently, in recent years, the concept and its impacts have garnered significant interest from researchers. More recently, numerous studies mainly concentrated on the influencing factors of adaptability and its effects on individuals. For instance, some studies have found that students' personality (Jiang, 2017), perceived social support (Z. Wang & Fu, 2015), and early parent–child relationship (Wibowo et al., 2020) have a significant impact on their adaptability in the future. Furthermore, several studies have discovered that adaptability is related to college students' academic achievement (Rebecca et al., 2017), coping style (Ma et al., 2022), life satisfaction (Ginevra et al., 2018), and mental health (Shin & Lee, 2019). Therefore, further investigating adaptability becomes essential due to its significant role in individual development.

Besides, several studies have indeed delved into the components of adaptability; however, different researchers hold varying perspectives on its constituent elements. For example, Soledad et al. (2012) identified four classifications of adaptability: learning adaptability, social adaptability, emotional adaptability, and dependence on universities. Campbell and Prichard (2000) classified adaptability into nine dimensions, revealing symptoms of maladaptive individuals, including anxiety, depression, suicidal ideation, substance abuse, low self‐esteem, interpersonal problems, family problems, career problems, and academic problems. Dam and Meulders (2020) proposed three dimensions for employee adaptability: cognitive adaptability, behavioral adaptability, and emotional adaptability. Erylmaz and Kara (2016) found that career adaptability consists of two components: career exploration and career planning. Cao and Mao (2008) developed one of the most popular measurements of adaptability in China (Cao & Luo, 2020; Lei & Zhou, 2017; Luo et al., 2021), comprising six dimensions: learning adaptability, professional adaptability, homesickness adaptability, interpersonal adaptability, emotional adaptability, and economic adaptability. Luo (2020) later simplified this scale into a 24‐item version based on the original scale.

Based on the literature review presented above, it becomes evident that adaptability is comprised of intricately interrelated constituent components. However, the relationships among these components appear to be quite complex, warranting further investigation. Two noteworthy points concerning the components of adaptability emerged from the literature. For one thing, some inconsistencies in the relationship between different dimensions of adaptability were revealed. For instance, some studies discovered significant correlations between adaptability dimensions, such as the correlations between emotional adaptability, academic adaptability, and studying adaptability being higher than 0.8, and the correlations between academic adaptability and studying adaptability being higher than 0.7 (Feldt et al., 2011). However, other studies found small correlation coefficients between certain dimensions (Luo et al., 2015), such as the correlations between economic adaptability and learning adaptability, and professional adaptability, being less than 0.1, and the correlations between homesickness adaptability and learning adaptability also being less than 0.1. These contradictory findings suggest that the latent psychological components of adaptability may not be a typical multidimensional structure. Consequently, it is important to find out more about the latent psychological components of adaptability.

Another thing was that there were some overlaps among different adaptability measures. Previous research has found that emotional adaptability is strongly related to other types of adaptability, such as academic adaptability (Huang et al., 2018), social adaptability (Feldt et al., 2011), and interpersonal adaptability (Zhang et al., 2022). Emotional adaptability seems to be a characteristic common across several adaptability measurements. Conspicuously, many studies found that emotional adaptability (such as positive and negative emotions, anxiety, and depression) was a key factor in determining adaptability (Chiu et al., 2020; Thorlacius & Gudmundsson, 2017). As a result, emotional adaptability was seen as the most important component in many adaptability measures (Zhang & Jiang, 2006).

Emotional adaptability has also been discussed in theories. According to the stress theory (Staal, 2004), specific stressors related to interpersonal adaptability, learning adaptability, professional adaptability, and economic adaptability could be identified easily, while there has not been a specific stressor directly associated with emotional adaptability. Some researchers have revealed that the existence of specific stressors may influence emotional adaptability strategies, leading to negative emotions (Acremont & Van der Linden, 2007). Furthermore, the Integrative Model proposed by Billieux (2012) claiming that individuals' maladaptive emotion regulation might be influenced by their maladjustment with other specific stressors, such as learning, professional, homesickness, interpersonal, and economic adaptability. This leads us to infer that emotional adaptability was a common symptom when students exhibited maladaptive performance in other types of adaptability. In other words, addressing students' maladjustments with specific stressors could potentially help improve their emotional adaptability. All in all, both theoretical studies and empirical results of adaptability indicated that emotional adaptability was a special dimension of adaptability that was not akin to or parallel to other dimensions of adaptability.

However, the majority of current research on adaptability relies on cross‐sectional data (Duchesne et al., 2007; Luo et al., 2021), which does not fully meet the requirements of this study that aims to examine the causal relationship between various dimensions of adaptability and emotional adaptability. Furthermore, adaptability has been recognized as a crucial trait characterized by dynamic attributes that individuals possess (Cao & Mao, 2008). For instance, Feng et al. (2006) discovered significant changes in learning adaptability, interpersonal adaptability, and emotional adaptability, and other studies revealed that college students demonstrated obvious improvement in academic and emotional adaptability over time, while interpersonal adaptability showed a decline (Cao & Mao, 2008; Xu et al., 2005). Consequently, this study has been structured as a longitudinal investigation, utilizing appropriate methods to address its specific research focus.

The primary objective of this research was to investigate the latent structure of adaptability, and it comprised two sub‐studies. In Study 1, the focus was on unravelling the latent structure of adaptability among college students using a cross‐sectional dataset. This investigation employed both confirmatory factor analysis (CFA) and structural equation modeling (SEM). The main aim of Study 1 was to assess the specificity of emotional adaptability. Subsequently, Study 2 was conducted to confirm the association between emotional adaptability and other types of adaptability. This validation was accomplished through the utilization of cross‐lagged panel models (CLPMs), an analytical method designed to explore causal relationships among multiple variables within a longitudinal dataset. Based on the stress theory and the theory of Billieux (2012), we hypothesized that (1) adaptability is not a simple first‐order structure and (2) emotional adaptability could be influenced by other types of adaptability, including learning, professional, homesickness, interpersonal, and economic adaptability.

## STUDY 1

### Method

#### Participants

A total of 578 volunteers from college and graduate school in Guizhou completed a 5‐min survey through paper–pencil questionnaires. However, 13 participants were removed due to lack of answering more than 10 items. Finally, the sample comprised 565 subjects, including 317 women (56%) and 248 men (44%). Participants' mean age was 19.09 years (range 16 to 23 years, *SD* = 1.01). All participants consented to the use of their data, and the study was approved by the Human Research Protection Committee of Tianjin Normal University in China (ethical review number: XL2020‐08).

#### Measurement

The Freshmen Adaptability Scale was developed by Cao and Mao (2008), and it was revised by Luo (2020). The revised version of the Freshmen Adaptability Scale was used in this research. The scale has 24 items, including learning, professional, homesickness, interpersonal, emotional, and economic adaptability. An example item is “I can guarantee the time for self‐study every day.” It is a 6‐point Likert scale, ranging from 1 (*extremely inconsistent*) to 6 (*extremely consistent*), with a higher score indicating a higher level of adaptability. Cronbach's *α* for the whole scale was .809, and for the six dimensions it ranged from .660 to .838 in Sample 1. Cronbach's *α* for the whole scale was .853; for the six dimensions it ranged from .750 to .888 in Sample 2; and the *ω* of the whole scale was .832.

#### Statistical methods

In this study, data preprocessing and reliability analysis were conducted using SPSS 26. Mplus 8.0 was employed for a range of analyses, including CFA, SEM, and CLPM. CFA was conducted to explore the structure of adaptability. Five SEMs were applied to investigate the relationship between emotional adaptability and other types of adaptability in the cross‐sectional datasets, while two types of CLPM were utilized to validate these relationships in longitudinal datasets. These two types of CLPMs differ in whether they include paths from emotional adaptability to other types of adaptability. The first type of model only includes paths from other types of adaptability to emotional adaptability, while the second type of model includes both paths from emotional adaptability to other types of adaptability and vice versa. Each type of CLPM comprises five models, corresponding to the five different adaptability dimensions. Model fit was assessed using the following indices, as suggested by Kline (2016) and Wen et al. (2004): the root‐mean‐square error of approximation (RMSEA), standardized root‐mean‐square residual (SRMR), comparative fit index (CFI), and Tucker–Lewis index (TLI). RMSEA and SRMR values should be below .08, while CFI and TLI values greater than .900 are considered acceptable, and values exceeding .950 indicate good model fit.

### Results

#### CFA model for adaptability

**FIGURE 1:**
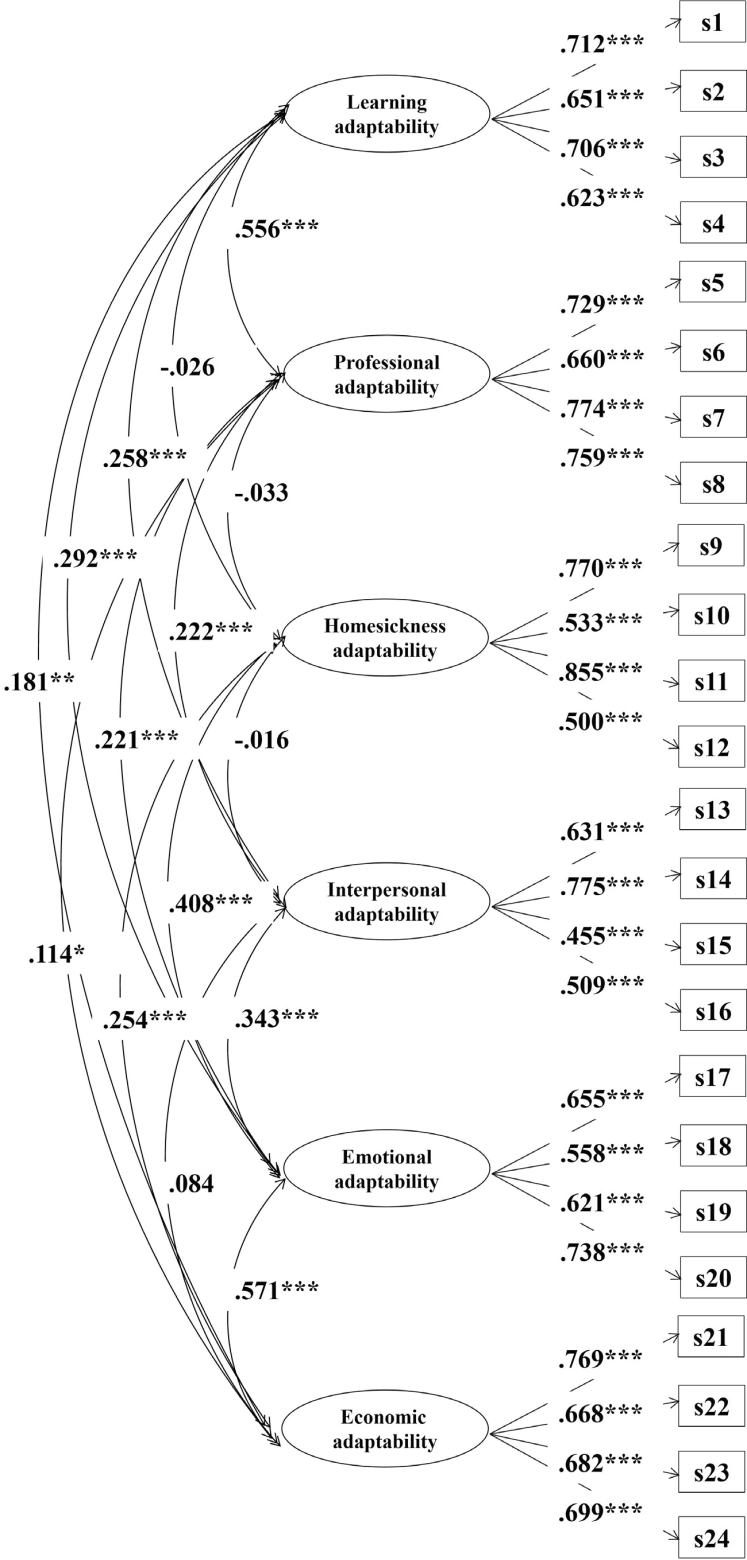
The confirmatory factor analysis (CFA) model of adaptability. ***p < .001, **p < .010, *p < .050.

First, CFA was conducted to verify the structure of adaptability. Good model fit (*χ* ^2^ = 606.935, *p* < .001), *df* = 234, TLI = .900, CFI = .915, SRMR = .054, RMSEA = .053) was demonstrated, and factor loadings ranged from .455 to .855. According to the results it can be found that two absolute fit indices (SRMR < .100, RMSEA < .100) and two relative fit indices (CFI > .900, TLI > .900) showed the CFA model of adaptability was acceptable. The CFA model can be seen in Figure 1.

Correlations between the latent variables of adaptability were discovered through the CFA model. The results revealed a higher correlation between learning adaptability and professional adaptability (*r* = .556, *p* < .001). Because they are both measuring individual academic problems, there was a higher correlation between them. However, in order to maintain as much adaptability as possible, the study still regarded them as different dimensions. Additional results revealed that emotional adaptability was significantly correlated with other types of adaptability, with correlations ranging from .221 to .571 (*p* < .001). But the correlations between them were very small (*r* coefficients were smaller than .26), and some coefficients were not significant at all (*r*s ranged from −.033 to .084, *p*s were higher than .050). Details can be seen in Table 1. Therefore, they were treated as separate dimensions in this research.

**TABLE 1:** Correlation among different latent variables of adaptability.

Variables	1	2	3	4	5	6
1 Learning adaptability	‐					
2 Professional adaptability	.556***	‐				
3 Homesickness adaptability	−.026	−.033	‐			
4 Interpersonal adaptability	.258***	.222***	−.016	‐		
5 Emotional adaptability	.292***	.221***	.408***	.343***	‐	
6 Economic adaptability	.181**	.114*	.254***	.084	.571***	‐

*** *p* < .001.

** *p* < .010.

* *p* < .050.

Above all, the results indicated that the uniqueness of emotional adaptability should be taken into account. As a consequence, the study further investigated whether emotional adaptability was a common manifestation of other types of adaptability. Thus, the latent correlation between other types of adaptability and emotional adaptability in the original model was turned into the predictive effect of emotional adaptability on other types of adaptability.

#### Latent psychological structure of adaptability

Second, five SEMs were done to investigate the effects of emotional adaptability on other types of adaptability, as these dimensions were regarded as separate components. According to the results, absolute fit indices (SRMR < .100, RMSEA < .100) and relative fit indices (CFI > .900, TLI > .900) showed five SEMs were acceptable. For example, good model fit (*χ* ^2^ = 77.514, *p* < .001, *df* = 19, TLI = .922, CFI = .947, SRMR = .048, RMSEA = .074) was demonstrated in the model of emotional adaptability and learning adaptability and good model fits were demonstrated in the other four SEMs. The results can be seen in Table 2. Results also showed that there were significant associations between emotional adaptability with learning adaptability (*β* = .280, *p* < .001), professional adaptability (*β* = .231, *p* < .001), homesickness adaptability (*β* = .412, *p* < .001), interpersonal adaptability (*β* = .338, *p* < .001), and economic adaptability (*β* = .588, *p* < .001).

**TABLE 2:** Structural equation modeling for the relationship between emotional adaptability on other types of adaptability.

Model	Model fit index							Prediction coefficient	
	*χ* ^2^	Sig.	*df*	CFI	TLI	RMSEA	SRMR	*β*	Sig.
Learning adaptability → Emotional adaptability	77.514	<.001	19	.947	.922	.074	.048	.280	<.001
Professional adaptability → Emotional adaptability	94.201	<.001	19	.948	.923	.084	.040	.231	<.001
Homesickness adaptability → Emotional adaptability	60.833	<.001	19	.964	.947	.062	.045	.412	<.001
Interpersonal adaptability → Emotional adaptability	59.262	<.001	19	.955	.934	.061	.035	.338	<.001
Economic adaptability → Emotional adaptability	78.825	<.001	19	.954	.933	.075	.039	.588	<.001

Abbreviations: CFI, comparative fit index; RMSEA, root‐mean‐square error of approximation; SRMR, standardized root‐mean‐square residual; TLI, Tucker–Lewis index.

## STUDY 2

### Method

#### Participants

Participants were recruited to complete an 8‐min longitudinal survey through a paper–pencil questionnaire. All the participants were college students in Tianjin, a large city in China. Paper and pencil measures were administered in classrooms to about 50 participants each time. All participants provided their written informed consent to participate in this research, and they completed the questionnaire. At the first wave in October, 2020, 494 volunteers completed the survey. Then, they filled out the same questionnaire again after 1 month, and they had to fill it out three times. A total of 402 subjects participated, including 68 men (16.9%) and 334 women (83.1%). Participants' mean age was 19.51 years (range 17 to 27 years, *SD* = 1.90).

#### Measurement and statistic method

The measurement and statistic method were the same as Study 1.

### Results

#### CLPMs of emotional adaptability on other types of adaptability

**FIGURE 2:**
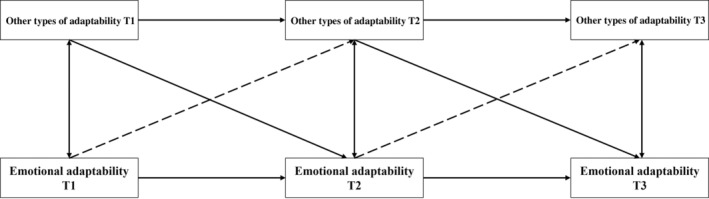
Two kinds of cross‐lagged panel model (CLPM) about emotional adaptability and other types of adaptability. Dashed lines represent paths exclusive to the second type of model, while solid lines depict paths present in both the first and second types of model.

Finally, two kinds of CLPMs were conducted to investigate the impact of emotional adaptability on other types of adaptability by comparing the two kinds of models. The main distinction between the two types of models lies in the presence of paths from emotional adaptability to other types of adaptability. The first type of model only includes the paths from other types of adaptability to emotional adaptability. In contrast, the second type of model encompasses both the paths from emotional adaptability to other types of adaptability and the paths from other types of adaptability to emotional adaptability. Given that the study focused on five kinds of types of adaptability, 10 CLPMs were constructed based on the longitude datasets in this study. For a more detailed representation of the model, please refer to Figure 2.

According to the model fit indicators, it can be found that first type of CLPM (*χ* ^2^ = 14.976, *p <* .010, *df* = 4, TLI = .943, CFI = .984, SRMR = .072, RMSEA = .083) was better than the second type of CLPM (*χ* ^2^ = 48.075, *p <* .001, *df* = 4, TLI = .722, CFI = .935, SRMR = .044, RMSEA = .166) in the relationship between emotional adaptability and learning adaptability. The Bayesian Information Criteria (BIC) value of the first type of CLPM (BIC = 5070.641) was smaller than the second type of CLPM (BIC = 5138.969). It also supported that the first type of CLPM fitted better in the relationship between emotional adaptability and learning adaptability. Similarly, the same results could be found in the relationship between emotional adaptability and professional, homesickness, interpersonal, and economic adaptability. The detailed results can be seen in Table 3.

**TABLE 3:** Two kinds of CLPM model fit indicators between the relationship between emotional adaptability and other types of adaptability.

Independent variables	First type of CLPM models	Fit indicators of the first type of CLPM							Second type of CLPM models	Fit indicators of second type of CLPM						
		BIC	*χ2*	df	CFI	TLI	SRMR	RMSEA		BIC	*χ*2	*df*	CFI	TLI	SRMR	RMSEA
Learning	Model A	5070.641	14.976	4	.984	.943	.072	.083	Model a	5138.969	48.075	4	.935	.772	.044	.166
Professional	Model B	5217.726	10.485	4	.994	.978	.040	.064	Model b	521.436	50.393	4	.955	.843	.036	.170
Homesickness	Model C	5363.214	8.426	4	.996	.985	.037	.052	Model c	5441.856	65.073	4	.942	.797	.037	.195
Interpersonal	Model D	4922.293	20.248	4	.978	.922	.067	.101	Model d	4970.315	48.326	4	.939	.788	.038	.166
Economic	Model E	5358.607	27.089	4	.975	.911	.096	.120	Model e	5417.953	65.755	4	.932	.761	.048	.196

*Note*: “Learning” denotes learning adaptability, “Professional” denotes professional adaptability, “Homesickness” denotes homesickness adaptability, “Interpersonal” denotes interpersonal adaptability, “Emotional” denotes emotional adaptability, and “Economic” denotes economic adaptability.

Abbreviations: BIC, Bayesian Information Criteria; CFI, comparative fit index; CLPM, cross‐lagged panel model; RMSEA, root‐mean‐square error of approximation; SRMR, standardized root‐mean‐square residual; TLI, Tucker‐Lewis index.

The summary of the first type of CLPM is displayed in Table 4. Results also showed that all of the auto‐regression paths were significant in five CLPMs (see Table 4). The variables measured at T1, including learning adaptability (*β* = .698, *p* < .001), professional adaptability (*β* = .825, *p* < .001), homesickness adaptability (*β* = .799, *p* < .001), interpersonal adaptability (*β* = .699, *p* < .001), economic adaptability (*β* = .648, *p* < .001), and emotional adaptability (*β* ∈ [.655, .702], *p* < .001), positively predicted themselves at T2. Similarly the variables measured at T2 could also positively predict themselves at T3 (see Table 4). The results demonstrated that learning adaptability, professional adaptability, homesickness adaptability, interpersonal adaptability, emotional adaptability, and economic adaptability were relatively stable over a 2‐month period.

**TABLE 4:** The first type of CLPM of emotional adaptability with different other types of adaptability.

First type of CLPM models	Path in first type of CLPM	*β*
Model A: Learning → Emotional	T1 Learning → T2 Emotional	.154***
	T2 Learning → T3 Emotional	.096*
	T1 Learning → T2 Learning	.698***
	T2 Learning → T3 Learning	.514***
	T1 Emotional → T2 Emotional	.663***
	T2 Emotional → T3 Emotional	.501***
Model B: Professional → Emotional	T1 Professional → T2 Emotional	.084*
	T2 Professional → T3 Emotional	.074
	T1 Professional → T2 Professional	.825***
	T2 Professional → T3 Professional	.649***
	T1 Emotional → T2 Emotional	.702***
	T2 Emotional → T3 Emotional	.480***
Model C: Homesickness → Emotional	T1 Homesickness → T2 Emotional	.034
	T2 Homesickness → T3 Emotional	.087*
	T1 Homesickness → T2 Homesickness	.799***
	T2 Homesickness → T3 Homesickness	.682***
	T1 Emotional → T2 Emotional	.707***
	T2 Emotional → T3 Emotional	.489***
Model D: Interpersonal → Emotional	T1 Interpersonal → T2 Emotional	.051
	T2 Interpersonal → T3 Emotional	.152***
	T1 Interpersonal → T2 Interpersonal	.699***
	T2 Interpersonal → T3 Interpersonal	.648***
	T1 Emotional → T2 Emotional	.686***
	T2 Emotional → T3 Emotional	.463***
Model E: Economic → Emotional	T1 Economic → T2 Emotional	.103*
	T2 Economic → T3 Emotional	.124**
	T1 Economic → T2 Economic	.648***
	T2 Economic → T3 Economic	.612***
	T1 Emotional → T2 Emotional	.655***
	T2 Emotional → T3 Emotional	.476***

*Note*: “Learning” denotes learning adaptability, “Professional” denotes professional adaptability, “Homesickness” denotes homesickness adaptability, “Interpersonal” denotes interpersonal adaptability, “Emotional” denotes emotional adaptability, and “Economic” denotes economic adaptability.

Abbreviation: CLPM, cross‐lagged panel model.

*** *p* < .001.

** *p* < .010.

* *p* < .050.

Additionally, results also showed the cross‐lagged paths in five CLPMs. The model of learning adaptability and emotional adaptability showed that learning adaptability at Time 1 positively predicted emotional adaptability at Time 2 (*β* = .154, *p* < .001), and the effect was significant from Time 2 to Time 3 (*β* = .096, *p* < .050). Also, there were significantly relationships between learning adaptability and emotional adaptability in the same timeframe, and the *r*‐values ranged from .134 to .297 (*p* < .010), except the relationship between learning adaptability and emotional adaptability in T3 (*r* = .037, *p* = .660). Similarly, CLPMs results of economic adaptability and emotional adaptability showed that the lagged path from T1 economic adaptability to T2 emotional adaptability was significant (*β* = .103, *p* < .050), and the lagged path from T2 economic adaptability to T3 emotional adaptability was also significant (*β* = .124, *p* < .010). It means that learning adaptability and economic adaptability could predict emotional adaptability.

Besides that, coefficient results in the professional adaptability and emotional adaptability CLPMs showed that that professional adaptability at Time 1 positively predicted emotional adaptability at Time 2 (*β* = .084, *p* < .050). The cross‐lagged path from T2 homesickness adaptability to T3 emotional adaptability was significant (*β* = .087, *p* < .050) in the CLPMs of homesickness adaptability and emotional adaptability. Furthermore, the coefficient results in the CLPMs of interpersonal adaptability and emotional adaptability showed that the lagged path from T2 interpersonal adaptability to T3 emotional adaptability was significant (*β* = .152, *p* < .001), which means that emotional adaptability could also be predicted by professional adaptability, homesickness adaptability, and interpersonal adaptability. Other results can be seen in Table 4.

## DISCUSSION

### Relationship between emotional adaptability and other types of adaptability

Are there any other adaptability determinants of emotional adaptability? This research utilized both cross‐sectional and longitudinal designs to examine the relationship between emotional adaptability and five kinds of adaptability among Chinese college students. The results demonstrated that emotional adaptability could be predicted by learning, professional, homesickness, interpersonal, and economic adaptability. Overall, this research represents a fundamental exploration of the connection between emotional adaptability and these five kinds of adaptability.

The research consists of two main parts. In Study 1, SEM was employed to explore the relationship between emotional adaptability and five types of adaptability. According to the results of Study 1, emotional adaptability was found to have distinct associations with other dimensions of adaptability, and specific relationships were observed between emotional adaptability and these dimensions (details in Tables 1 and 2). These findings align with previous research conducted by Huang et al. (2018), where they also discovered that college students' adaptability in professional, learning, homesickness, interpersonal, and economic domains was significantly correlated to emotional adaptability. Besides, this conclusion has been supported by other investigations (Wang & Liu, 2021).

Furthermore, in Study 2, using a longitudinal dataset, the links between emotional adaptability and other types of adaptability were validated. The results revealed that emotional adaptability was strongly influenced by college students' learning, professional, homesickness, interpersonal, and economic adaptability (see Table 4). These findings provide support for the stress theory hypothesis (Staal, 2004) and Billieux's (2012) integrative model, which claimed that individuals' emotional maladjustment without a specific stressor can be influenced by other cognitive maladaptation and behavioral problems with specific stressors (Igarashi et al., 2008; Masoud et al., 2021). This study identifies significant implications for schools and educators based on its findings. For instance, if they discover college students with poor emotional adaptability, they may investigate whether the students' negative emotional issues are due to academic, financial, or homesickness‐related factors. College students, notably freshmen, will encounter a variety of challenges, including difficulties in socializing with their classmates and the problems of adapting to an autonomous learning environment. It was suggested that universities should focus on reducing students' stressors by organizing more seminars for freshmen and more extracurricular activities to assist them in overcoming these challenges and thereby alleviating their emotional dysregulation.

Notably, this research did not find any significant bicorrelation between each dimension of adaptability. For instance, the correlations between homesickness adaptability and learning adaptability (*r* = .026, *p* > .050), professional adaptability (*r* = .033, *p* > .050), and so on, showed no significant associations. This outcome was consistent with findings from other studies (Luo et al., 2015; Wang et al., 2010), suggesting that not all types of adaptability were correlated with each other (Taylor & Pastor, 2007). In other words, maladjustment in one aspect among college students did not necessarily occur across all aspects.

### Implications

The present study focuses on investigating the relationship between emotional adaptability and five other types of adaptability, yielding several significant implications. First, it goes beyond merely examining the relationship through a cross‐sectional dataset and employs a longitudinal study to validate the impact of emotional adaptability on other types of adaptability. Consequently, this study provided a comprehensive understanding of emotional adaptability and its association with other types of adaptability. Moreover, the present study utilized two distinct types of cross‐lagged panel analysis models to reveal a unidirectional influence, whereby other facets of adaptability influence emotional adaptability. This discovery marks a notable advancement within the field of adaptability research. Third, the research offers compelling evidence that supports the assumptions of the stress theory (Staal, 2004) and the integrative model (Billieux, 2012) by utilizing empirical data. It demonstrates that emotional adaptability is a common manifestation of maladaptive problems with specific stressors. That is, students' maladjustment in learning, social interactions with classmates, homesickness, and economic difficulties may trigger emotional dysregulation (Masoud et al., 2021). Fourth, considering adaptability is a pivotal cognitive trait (Gerald, 2018), this study deepened our understanding of the connection between other forms of adaptability and emotional adaptability. It highlighted that emotional maladjustment can be linked to learning, professional pursuits, interpersonal relationships, economic problems, or it can arise from homesickness maladjustment. Consequently, if universities aim to enhance college students' emotional adaptability, interventions should focus on alleviating the pressure associated with these specific types of adaptability (Turgeman‐Lupo et al., 2022). Thus, in turn, they can enhance their cognitive traits and contribute to higher life satisfaction.

### Limitations and future work

Nevertheless, some limitations of this study should be noted. First, the present study just conducted a questionnaire survey to reveal the relationship between different components of adaptability, showing that college students' learning, professional, homesickness, interpersonal, and economic maladjustments could influence the level of emotional maladjustments significantly. Therefore, we hope to provide psychological suggestions for individuals who suffer from maladaptive problems by conducting clinical or intervention experiments in future studies. Second, the sample sizes of 565 and 402 college students may need to be larger to generalize the findings to the entire college student population. To address this limitation, we plan to recruit a larger sample size in future studies to enhance the stability and reliability of our results. Besides that, the results of this study are specific to a Chinese context. Therefore, we hope to conduct an intercultural study to confirm the results in a Western population. Fourth, the psychological mechanisms underlying the predictive role of five kinds of adaptability in emotional adaptability need further exploration. For example, we need to incorporate other personality traits into our model and control the influence of other variables.

## CONCLUSION

The current study revealed that adaptability is not a simple first‐order structure and includes learning adaptability, professional adaptability, homesickness adaptability, interpersonal adaptability, emotional adaptability, and economic adaptability. In addition, this study also found that an individual's emotional adaptability could be predicted by other types of adaptability by using a combination of cross‐sectional and longitudinal datasets. That is, individuals would exhibit a commonality of emotional maladjustment if they have maladaptive problems in learning, homesickness, interpersonal, or economic adaptability.

## AUTHOR CONTRIBUTIONS

Shixiu Ren collecting the dataset, analysis and interpretation of data, writing – original draft, writing – review & editing; Shunxin Ji, collecting the dataset; Xinyang Liu, collecting the dataset; Tour Liu, acquisition of data, writing – review & editing, final approval of the version to be published.

## FUNDING INFORMATION

This study were funded by National Natural Science Foundation of China (grant 31800945) and Graduate Independent Project of the Collaborative Innovation Center of Assessment for Basic Education Quality, Beijing Normal University (grant BJZK‐2021A1‐20013).

## CONFLICT OF INTEREST STATEMENT

The authors declare they have no conflict of interest.

## ETHICAL STATEMENT

All procedures followed were in accordance with the ethical standards of the responsible committee on human experimentation (Tianjin Normal University, China) and with the Helsinki Declaration of 1975, as revised in 2000 (5) (Ethical review number: XL2020‐08).

## INFORMED CONSENT

We informed the participants about the relevant content of the study before the test, and obtained the participants' consent. We also informed the participants that the data would only be used for scientific research, and that they have the right to automatically withdraw at any time.

## Data Availability

The data that support the findings of this study are available from the corresponding author upon reasonable request.

## APPENDIX A

This section presents the items of the Freshman Adaptability Scale. The scale contains six dimensions: learning, professional, homesickness, interpersonal, emotional, and economic adaptability, and includes a total of 24 items, with four items in each dimension. “Learning adaptability” denotes the psychological and behavioral capacity to adapt and harmonize one's learning behaviors in response to learning requirements and environmental pressures (Feng et al., 2006). “Professional adaptability” refers to the process of refining one's professional comprehension through engagement with one's study major and professional environment, ultimately fostering professional growth (Wang et al., 2010). “Interpersonal adaptability” involves the skill of managing the interpersonal and societal demands of college life (Soledad et al., 2012). “Emotional adaptability” refers to a person's psychological state and their ability to manage emotional distress (Soledad et al., 2012). “Homesickness adaptability” is defined as the distress or impairment caused by an actual or anticipated separation from home (Thurber & Walton, 2012). Economic adaptability refers to the psychological and behavioral capacity to adjust and balance consumption behavior and emotional state in response to economic circumstances (Guo et al., 2011) Table A1.

**TABLE A1:** Twenty‐four items of the Freshman Adaptability Scale.

Dimension	Items
Learning adaptability:	Item 05. I can manage time effectively. Item 10. I am able to concentrate on listening to the class. Item 12. I can guarantee the time for self‐study every day. Item 18. My learning goals are clear.
Professional adaptability	Item 01. I like my major. Item 06. I am interested in professional learning. Item 19. My major helps me find my ideal career. Item 23. I like the development prospects of my major.
Homesickness adaptability	*Item 02. I missed home a lot after entering university. *Item 07. I am dying for home‐cooked food. *Item 17. I really want to go home during the period at university. *Item 20. I often look through the photos of my family.
Interpersonal adaptability	Item 03. I get along well with my roommates. Item 13. I get along well with my classmates. Item 21. I like the group life in the university dormitory. Item 24. I do not think it is difficult to get along with college classmates.
Emotional adaptability	*Item 08. I feel lonely at university. *Item 11. My mood is very impetuous. *Item 15. I have low self‐esteem in college. *Item 22. I had an inexplicable sense of fear after entering college.
Economic adaptability	*Item 04. I do not have any money left except for boarding fee. *Item 09. I was caught off guard by the high consumption in university. *Item 14. Due to financial reasons, I seldom bought new clothes after entering school. *Item 16. I am worried that my living expenses will not be paid in the future.

*Note*: *Reversed item.
